# Zero-Dose Children, Misinformation, and Vaccine Hesitancy

**DOI:** 10.7759/cureus.82028

**Published:** 2025-04-10

**Authors:** Abdulqadir J Nashwan, Sawsan Abuhammad

**Affiliations:** 1 Nursing & Midwifery Research Department, Hamad Medical Corporation, Doha, QAT; 2 Department of Nursing, College of Health Sciences, University of Sharjah, Sharjah, ARE

**Keywords:** immunization, misinformation, public health, vaccine hesitancy, zero-dose children

## Abstract

This editorial addresses the urgent and multifaceted issue of zero-dose children - those who have not received any routine vaccinations, such as for measles, diphtheria, or polio. These children are predominantly found in marginalized communities, conflict zones, and areas with limited healthcare infrastructure. The rise in zero-dose cases is driven by a combination of misinformation, vaccine hesitancy, poverty, geographic inaccessibility, gender-based barriers, and historical mistrust in healthcare systems. The recent resurgence of vaccine-preventable diseases highlights the need for immediate and collaborative action. The authors advocate for integrated solutions, including the engagement of community and religious leaders, digital platform accountability, and inclusive public health policies. Successful case studies - such as France and Italy’s mandatory vaccination laws, Brazil’s Bolsa Família program, and mobile health initiatives - demonstrate effective pathways to increase vaccine coverage. Rebuilding trust through culturally sensitive education, transparency, and improved access to immunization services is essential to ensure that no child is left behind.

## Editorial

There is a relatively new and alarming phenomenon known as zero-dose children [1]. These children have not received a single dose of any routine childhood vaccine such as those for measles, diphtheria, or polio - placing them at high risk for vaccine-preventable diseases [1]. Typically, these children are found in marginalized communities, conflict zones, vaccine-hesitant households, and areas with limited or fragile healthcare infrastructure [2].

The United Nations International Children’s Emergency Fund (UNICEF) and the World Health Organization (WHO) reported in 2023 that around 14.3 million children worldwide remain zero-dose due to a range of factors, including poverty, geographic inaccessibility, gender-based barriers, and systemic inequalities [3]. These are not only structural barriers but also social and psychological challenges, such as widespread misinformation about the safety and efficacy of vaccines [4].

A major emerging driver of vaccine hesitancy is the spread of misinformation through social media platforms, messaging apps, and word-of-mouth communication [5]. Parents exposed to misleading claims - such as vaccines causing autism, infertility, or other severe side effects - may become fearful and reluctant to immunize their children [6]. In some communities, these fears are compounded by deeply rooted mistrust in healthcare systems. Misconceptions, including conspiracy theories that portray vaccines as tools for government surveillance or population control, have continued to influence public perception - particularly in historically underserved or marginalized populations [7,8]. Rather than portraying parents as “guided by conspiracy theories,” it is important to recognize that such beliefs often stem from exposure to misinformation and underlying anxieties shaped by past injustices or lack of healthcare transparency.

Cultural and religious beliefs further influence vaccine acceptance. For example, some communities express concern that vaccines contain ingredients that violate religious dietary laws or that immunization interferes with divine will [9]. In patriarchal or conservative households, skepticism from male leaders often leads to children being denied vaccines [10]. Additionally, past unethical medical experiments on marginalized populations have left a lingering mistrust in public health systems [11]. This distrust, still evident in many African and Indigenous communities, continues to shape attitudes toward modern healthcare interventions, including vaccination [12].

These intersecting factors have led to a resurgence of diseases previously under control. The rise in measles cases in parts of Europe and North America and renewed polio detections in areas once considered polio-free illustrate the devastating consequences of declining vaccination coverage [13,14]. The COVID-19 pandemic further exacerbated vaccine hesitancy, especially due to misinformation about mRNA vaccines (which use messenger RNA to instruct cells to produce a protein that triggers an immune response) and other newly developed formulations [15]. Following the pandemic, many parents began delaying or refusing routine vaccinations for their children- not only due to growing hesitancy but also due to limited access to healthcare facilities - contributing to the resurgence of preventable illnesses [16-17].

A multifaceted approach is essential to reversing this trend. Community engagement through trusted local leaders, religious figures, and healthcare professionals remains critical to rebuilding trust and addressing context-specific concerns [18]. Complementing this are policy-level interventions. For example, France and Italy have successfully implemented mandatory childhood vaccination laws, while Brazil’s Bolsa Família program links welfare benefits to child immunization status, boosting uptake in underserved areas [19,20]. In India, the Mission Indradhanush campaign has enhanced access to routine immunization through targeted outreach, and UNICEF’s U-Report has empowered youth and communities to access accurate vaccine information [18]. These efforts highlight the power of combining grassroots outreach with systemic incentives and legislative support.

Digital accountability must also be prioritized. Social media platforms should actively monitor and counter harmful misinformation while elevating verified public health content. Immediate, authoritative responses to circulating falsehoods are crucial in mitigating their spread [21]. Governments must also strengthen immunization policies by ensuring vaccines are widely accessible, integrated into school enrollment, and supported by gender-sensitive and culturally appropriate delivery systems [22]. Mobile health initiatives, particularly in hard-to-reach regions, offer a promising solution to reduce logistical barriers and bring vaccines directly to needy families [23].

To sum up, addressing vaccine misinformation and hesitancy is essential to reducing the number of zero-dose children, but this alone is not enough. Structural barriers such as poverty, geographic inaccessibility, gender-based restrictions, and limited healthcare infrastructure should also be tackled to ensure equitable access to routine immunizations. The global community must work to rebuild trust in vaccines through education, transparency, and inclusive, well-resourced healthcare policies. Millions of vulnerable children can be protected from life-threatening diseases by prioritizing accurate information and strengthening childhood immunization programs.
